# Supplementary figures and images for: Reversal of pathological motor behavior in a model of Parkinson’s disease by striatal dopamine uncaging
Source: PLoS One. 2023 Aug 18;18(8):e0290317. doi: 10.1371/journal.pone.0290317 (PMC10437883; doi:10.1371/journal.pone.0290317)

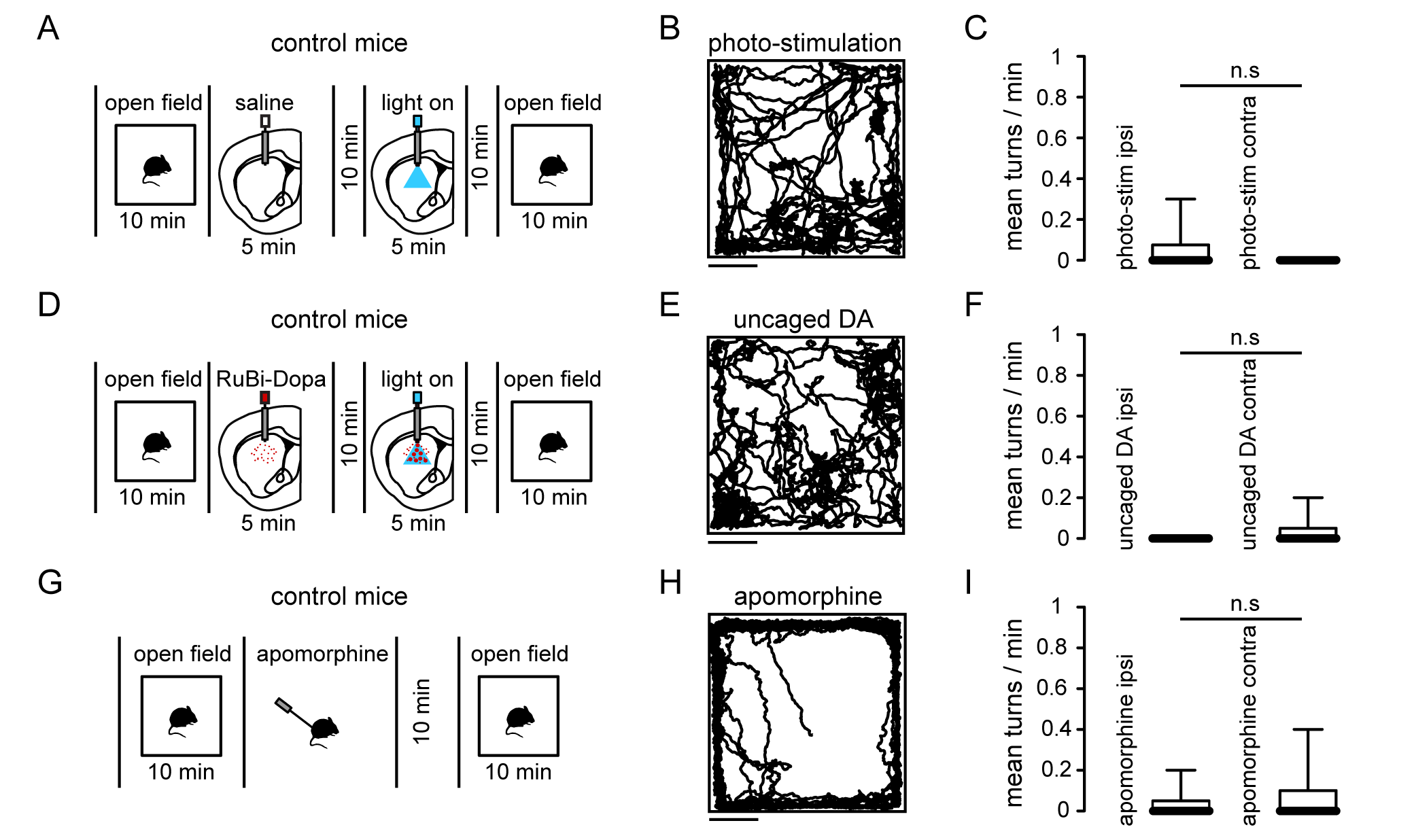

Supplement: S1 Fig — (A) Experimental timeline of unilateral striatal photo-stimulation in control mice. (B) Movement trajectory of a representative control mouse placed on an open field arena after striatal injection of saline followed by photo-stimulation. Scale bar: 10cm. (C) Striatal photo-stimulation in control mice doesn’t evoke contralateral turning behavior. (D) Experimental timeline of unilateral striatal injection of RuBi-Dopa in control mice. (E) Movement trajectory of a representative control mouse placed on an open field arena after striatal dopamine uncaging. Scale bar: 10cm. (F) Dopamine uncaging in control mice doesn’t evoke contralateral turning behavior. (G) Experimental timeline of systemically injected apomorphine in control mice. (H) Movement trajectory of a representative control mouse placed on an open field arena after systemic injection of apomorphine. Scale bar: 10cm. Note that the mouse moves close to the border of the open field arena. (I) Systemic injection of apomorphine in control mice doesn’t evoke contralateral turning behavior. (TIF) [file pone.0290317.s008.tif]
