# Supplementary material for: Reversal of pathological motor behavior in a model of Parkinson’s disease by striatal dopamine uncaging
Source: PLoS One. 2023 Aug 18;18(8):e0290317. doi: 10.1371/journal.pone.0290317 (PMC10437883; doi:10.1371/journal.pone.0290317)
Supplement: Attachment — Submitted filename: Comments (PONE-D-23-15033).docx [file pone.0290317.s010.docx]

**Manuscript Title-** Reversal of pathological motor behavior in a model of Parkinson’s disease by dopamine uncaging

**Manuscript Id-** PONE-D-23-15033

In the present study, the authors reported that stereotaxic administration of caged molecule RuBi-Dopa and its irradiation by blue light resulted in controlled increase in the striatal dopamine level and reduced the contralateral turning behavior in 6-OHDA induced PD mice.

The present piece of work appears interesting and might make significant contribution in the development of smart and cutting-edge treatment strategies for PD. However, some issues have been noticed which require modifications before further consideration and acceptance.

**Comments-**

1. The abstract appears to be unnecessarily long and does not contain concise and wholesome information about the entire work. The abstract must be modified in such a way that readers do not feel off-track while reading it. The abstract must contain a wholesome gist of introduction, methodology, results, and final conclusion and future perspective.
2. In the Introduction section, the authors must write more about caged molecules, their physical and chemical aspects, working mechanism, and their contribution so far in the field of medicine and PD.
3. The figures provided require proper alignment.
4. The authors must provide more information about what plausible side effects or inconvenience to the recipients this presented approach of PD treatment can bring.
5. The novelty of the present work must be highlighted in a more justifiable way.
